# Enhancing Radar Echo Extrapolation by ConvLSTM2D for Precipitation Nowcasting

**DOI:** 10.3390/s24020459

**Published:** 2024-01-11

**Authors:** Farah Naz, Lei She, Muhammad Sinan, Jie Shao

**Affiliations:** 1 School of Computer Science and Engineering, University of Electronic Science and Technology of China, Chengdu 611731, China; farahnaz@std.uestc.edu.cn (F.N.); leishe519@163.com (L.S.); sinan@std.uestc.edu.cn (M.S.); 2 Sichuan Artificial Intelligence Research Institute, Yibin 644000, China

**Keywords:** precipitation nowcasting, radar echo, spatiotemporal dynamics

## Abstract

Precipitation nowcasting in real-time is a challenging task that demands accurate and current data from multiple sources. Despite various approaches proposed by researchers to address this challenge, models such as the interaction-based dual attention LSTM (IDA-LSTM) face limitations, particularly in radar echo extrapolation. These limitations include higher computational costs and resource requirements. Moreover, the fixed kernel size across layers in these models restricts their ability to extract global features, focusing more on local representations. To address these issues, this study introduces an enhanced convolutional long short-term 2D (ConvLSTM2D) based architecture for precipitation nowcasting. The proposed approach includes time-distributed layers that enable parallel Conv2D operations on each image input, enabling effective analysis of spatial patterns. Following this, ConvLSTM2D is applied to capture spatiotemporal features, which improves the model’s forecasting skills and computational efficacy. The performance evaluation employs a real-world weather dataset benchmarked against established techniques, with metrics including the Heidke skill score (HSS), critical success index (CSI), mean absolute error (MAE), and structural similarity index (SSIM). ConvLSTM2D demonstrates superior performance, achieving an HSS of 0.5493, a CSI of 0.5035, and an SSIM of 0.3847. Notably, a lower MAE of 11.16 further indicates the model’s precision in predicting precipitation.

## 1. Introduction

The art and science of precipitation nowcasting serve an important role in various aspects of daily life, e.g., guiding individuals in planning their activities, ensuring appropriate attire, securing transportation, and safeguarding against outdoor hazards. Moreover, it plays an important role in optimizing agricultural practices, helping farmers in crop production, irrigation, and pest control [1]. Precipitation nowcasting is a forecasting method predicting imminent precipitation within a specified area, typically over the course of one to six hours. This technique is vital for planning and crisis management, as it offers critical direction for various tasks. Numerical weather prediction models are useful for longer-term forecasts, but they require high computational resources and time, making their use limited for urgent applications [2]. Observations from weather radars provide echo maps that are notable for their high spatial and temporal resolution. In modern precipitation nowcasting, the technique of extrapolating radar echo data from interconnected radars serves as a fundamental data source [3]. The challenge of precipitation nowcasting with radar imagery is conceptualized as a spatiotemporal prediction problem, aiming to understand image characteristics for precise forecasting.

There are two categories of current radar echo extrapolation techniques: optical flow-based models and deep learning-based algorithms [4,5,6]. Optical flow models estimate a motion field between successive maps, assuming constant pixel brightness. Extrapolation is then performed using this motion field. However, this assumption may not always hold, as the intensity of echo maps often changes, either strengthening or weakening. Moreover, these models usually only incorporate a few recent radar images, which restricts their ability to leverage valuable historical data. In general, the deep learning-based extrapolation methods perform better than optical flow approaches, as they are not constrained by the unrealistic assumption of constant brightness and can make effective use of past observations, leading to more accurate predictions [7].

The deep learning (DL) model can handle diverse data sources, understand complex correlations, and also simulate physical processes or build data-driven weather models. DL includes various algorithms such as artificial neural networks (ANNs) [8], CNNs [9], recurrent neural networks (RNNs) [10], support vector machines (SVMs) [11], random forests (RFs) [12], k-means clustering [13], and principal component analysis (PCA). However, it can overfit, underfit, lack interpretability, and require large data sets. RNNs are employed to identify temporal characteristics, whereas CNNs are commonly utilized for analyzing spatial attributes. Building on the diverse applications of deep learning in weather analysis, the focus will be on specialized models such as DA-LSTM, IDA-LSTM, E3D-LSTM, PredRNN, and PredRNN++ designed specifically for precipitation nowcasting using radar echo extrapolation.

The DA-LSTM (dual attention long short-term memory) model is a kind of RNN architecture, which is used in the field of radar echo extrapolation. It combines a dual attention mechanism that incorporates both temporal and spatial attention. Furthermore, the IDA-LSTM (interaction dual attention-LSTM) mechanism [14] takes a step further by combining an interaction system for short-term dependencies and a dual attention mechanism that concentrates on long-term temporal and channel information. This allows IDA-LSTM to capture more complex patterns in the data. Additionally, the E3D-LSTM (eidetic 3D-LSTM) model [15] is a state-of-the-art approach in spatiotemporal predictive modeling. Its three-dimensional extension of the conventional LSTM design allows it to capture complex long-term dependencies effectively. This expertise extends to a wide range of applications, including precipitation forecasting, traffic forecasting, and video prediction. Moreover, the PredRNN model [16] is a customized neural network designed for spatiotemporal predictive tasks. It combines spatial convolution layers with LSTM, enhancing its ability to capture temporal dynamics and spatial correlations at the same time, which makes it extremely useful for precipitation nowcasting. Its advanced version is PredRNN++ [17], which is built upon the PredRNN architecture by introducing causal LSTM units and a gradient highway unit (GHU).

Nonetheless, these models face various challenges in radar echo extrapolation. For instance, models such as the interaction-based dual attention LSTM have inherent limitations, including higher computational costs and resource requirements. Another noteworthy limitation is the consistent use of a fixed kernel size across layers, which hampers their ability to extract features globally and instead emphasizes local representations.

To address these challenges, we introduce convolutional long short-term memory 2D (ConvLSTM2D) based architecture for precipitation nowcasting. Our proposed method incorporates time-distributed layers. This approach allows for parallel convolutional-2D (Conv2D) operations on each image input, efficiently analyzing spatial patterns. Subsequently, ConvLSTM2D is applied to discern spatiotemporal features. The model operates on a multi-image basis, processing a sequence of five images to forecast precipitation 30 min ahead. It then utilizes the initially predicted 30 min precipitation data as input for subsequent 30 min predictions. The initial and final predictions are then concatenated along the sequence axis to create a comprehensive 60 min output sequence. Additionally, it employs varied kernel sizes, enabling the capture of both local and global features in spatiotemporal data, thereby enhancing computational efficiency and maintaining nowcasting accuracy. The evaluation metrics including the structural similarity index (SSIM), critical success index (CSI), mean absolute error (MAE), and Heidke skill score (HSS) are used to evaluate the efficacy of ConvLSTM2D relative to other algorithms.

Our technical contributions are as follows:

- We introduce ConvLSTM2D, a dual-component neural network architecture tailored for precipitation nowcasting. The proposed model incorporates ConvLayers, which are effective in extracting spatial and temporal features through the use of Conv2D and ConvLSTM2D operations.
- In previous studies, the limitation of fixed kernel sizes constrains models to local feature extraction. To address this, we explore multiple combinations of time-distributed Conv2D and ConvLSTM2D layers. The deliberate utilization of mixed kernel sizes enables the model to effectively capture both detailed information and broader spatial relationships.
- The performance of the ConvLSTM2D model is comprehensively assessed, demonstrating competitive outcomes through the use of metrics including CSI, HSS, SSIM, and MAE. The model’s predictability and accuracy in precipitation nowcasting is compared against the state-of-the-art models in the field.

## 2. Related Work

Radar echo extrapolation tasks in the context of precipitation nowcasting can be considered a type of spatiotemporal sequence problem. Currently, many researchers have employed deep neural networks for their investigations in this area [18,19,20]. In the field of precipitation nowcasting using radar echo data, there has been significant research progress recently. In [21], the ST-CNN network is used for extracting precursor information from data for extreme precipitation forecasting. Agrawal et al. [22] utilize a U-Net model to forecast rainfall status over short periods. Building on the U-Net framework, another study [23] introduces RainNet for predicting precipitation intensity five minutes ahead. Han et al. [24] employ a custom-designed loss function to train U-Net to predict radar echo images in the northern part of China 30 min in advance. RNN-based models are frequently integrated with convolutional layers to enhance their capacity for capturing spatial characteristics in radar echo datasets. This approach is exemplified in [25], where ConvLSTM is utilized for radar echo extrapolation. This research also demonstrates the conversion of predicted radar echoes into precipitation intensity.

Further advancing this domain, Shi et al. [26] introduce the TrajGRU unit, which outperforms their previous work [25]. To concurrently capture both temporal and spatial features, the PredRNN model [16] is developed, employing a novel spatiotemporal LSTM (ST-LSTM). E3D-LSTM [15] presents a model designed for spatiotemporal tasks, including precipitation forecasting, traffic forecasting, and human motion prediction. It extends the LSTM architecture to three dimensions, effectively capturing long-term dependencies and excelling in video prediction tasks. Addressing the issue of gradient disappearance noted in [16], Wang et al. develop the PredRNN++ model, as presented in [17]. This model incorporates multiple causal LSTM units along with a gradient highway (GHU), enhancing its computational efficiency. In addition, Luo et al. [14] introduce the IDA-LSTM model, which enhances radar echo extrapolation using interaction dual attention. It incorporates two novel elements: an interaction system for short-term dependency and a dual attention mechanism for long-term temporal and channel information. Empirical analysis on the CIKM AnalytiCup 2017 dataset demonstrates IDA-LSTM’s superior forecasting accuracy. To improve the accuracy in modeling radar echo motion, Yang et al. [27] improve ST-LSTM with a self-attention mechanism and extra memory. This advancement, creating the self-attention integrated ST-LSTM (SAST-LSTM) unit can effectively capture global spatial and temporal features of radar echoes. In 2021, a novel UNet-based model called Broad-UNet [28] is developed for deep learning-based weather nowcasting. This model improves feature extraction by replacing convolution layers with asymmetric parallel convolutions and adding atrous spatial pyramid pooling (ASPP) in pooling layers. The Pred-SF model, proposed in [29], predicts precipitation using a two-step process with multi-modal meteorological data. The focus of this study is mainly on extracting spatial features in the targeted areas. Ionescu et al. [30] propose DeePS, a family of CNN architectures, which makes a significant contribution to short-term weather nowcasting for meteorologists with a large volume of satellite and radar images. In response to a gap in numerical weather prediction methods for short-term forecasting, a new neural network called the small attention UNet (SmaAt-UNet) is introduced by Trebing et al. [18]. SmaAt-UNet achieves comparable performance using only 25% of the trainable network parameters. Another study based on the U-net architecture for precipitation nowcasting is LSTMATU-Net [31]. The Convolutional LSTM is integrated with U-Net with efficient channel and space attention module to address the loss of detail in predicted images.

## 3. Preliminaries

### 3.1. Mathematical Problem Formulation of Precipitation Nowcasting

The aim of precipitation nowcasting is to predict future radar maps within a local region using the observed radar echo sequence from the past. Weather radar records maps every 6 to 10 min in real-world scenarios, while nowcasting forecasts for the next 1 to 6 h, or 6 to 60 frames ahead of time. This problem can be viewed as a spatiotemporal sequence forecasting problem from a machine-learning perspective. In a scenario where we monitor a dynamic system across an X×Y grid, with each grid cell containing *Z* measurements that evolve over time, our observations at any given moment can be represented by a tensor $S\in R^{X\times Y\times Z}$ where *R* signifies the feature domain. When we collect these observations at regular intervals, we create a sequence of tensors $\hat{S}1,\;\hat{S}2,\;\ldots ,\;{\hat{S}}_{t}$. The spatiotemporal sequence forecasting problem involves predicting the most probable future sequence of length *N*, considering the *K* previous observations, which include the current one.

(1) $${\hat{S}}_{t+1},\;\ldots ,\;{\hat{S}}_{t+N}=\underset{S_{t+1},\;\ldots ,\;S_{t+N}}{\arg\;\max}p\left(S_{t+1},\;\ldots ,\;S_{t+N}|{\hat{S}}_{t-K+1},\;{\hat{S}}_{t-K+2},\;\ldots ,\;{\hat{S}}_{t}\right).$$

### 3.2. Long Short-Term Memory in Sequence Modeling

Long short-term memory (LSTM) networks have found applications in dynamic system modeling across various domains. A fundamental aspect of LSTM is the memory cell, denoted as $C_{t}$, which serves as an accumulator of state information [32,33]. Each memory cell is equipped with an internal state and several multiplicative gates, including the input gate ($i_{t}$), forget gate ($f_{t}$), and output gate ($o_{t}$). The input gate ($i_{t}$) determines whether incoming data should influence the internal state, while the forget gate ($f_{t}$) governs whether the previous cell state ($C_{t-1}$) should be retained or cleared. The output gate ($o_{t}$) dictates whether the internal state of a neuron should contribute to the cell’s output ($H_{t}$). The mathematical formulation for the single time step is given below:

(2) $$\begin{array}{cc} i_{t} & =\sigma (X_{t}W_{xi}+H_{t-1}W_{hi}+b_{i}), \end{array}$$

(3) $$\begin{array}{cc} f_{t} & =\sigma (X_{t}W_{xf}+H_{t-1}W_{hf}+b_{f}), \end{array}$$

(4) $$\begin{array}{cc} C_{t} & =f_{t}⊙C_{t-1}+i_{t}⊙\tanh\left(X_{t}W_{xg}+H_{t-1}W_{hg}+b_{g}\right), \end{array}$$

(5) $$\begin{array}{cc} o_{t} & =\sigma (X_{t}W_{xo}+H_{t-1}W_{ho}+b_{o}), \end{array}$$

(6) $$\begin{array}{cc} H_{t} & =o_{t}⊙\tanh\left(C_{t}\right), \end{array}$$

where $W_{xi}$, $W_{hi}$, $W_{xf}$, $W_{hf}$, $W_{xg}$, $W_{hg}$, $W_{xo}$ and $W_{ho}$ are the weight matrices, while $b_{i}$, $b_{f}$, $b_{g}$ and $b_{o}$ are the biases. The equations provided pertain to a single time step. Consequently, it is necessary to recalculate these equations for each subsequent time step in a sequence. In the case of a sequence consisting of $N_{t}$ time steps, the mentioned equations will be recalculated $N_{t}$ times for each time step, respectively.

## 4. Methodology

In this section, we introduce the proposed ConvLSTM2D, an extension of the LSTM model. Our proposed model utilizes the ConvLayers function, which defines a sequence of layers to process input sequences. These layers consist of Conv2D and ConvLSTM2D, which are widely employed in deep learning for image and sequence data analysis. The Conv2D layer is time-distributed, allowing parallel operations on each input image, thereby enhancing the analysis of spatial patterns. Assume that input sequence $X_{t}$ has the shape (M,T,H,W,C), where *M* is the number of samples, and *T* represents time step, *H* and *W* are the height and width of the image, respectively, and *C* is the number of channels in the input data. We suppose that there are *F* numbers of filters with kernel size $K_{H}\times K_{W}$ for each time step in this layer. The mathematical expression for the operation of Conv2D operation at position (i,j) of the input sequence at time step *t* for each filter *f* is given as follows:

(7) $$X_{t,f,i,j}^{\prime }=\sum_{l=0}^{K_{H}-1}\sum_{m=0}^{K_{W}-1}\sum_{n=0}^{C-1}\left(W_{f,l,m,n}.X_{t,s.i+l-p,s.j+m-p,n}\right)+B_{f},$$

where $X_{t,f,i,j}^{\prime }$ is the output of the one filter at position (i,j) of input sequence for time step *t*; $W_{f,l,m,n}$ is the weight tensor for filter *f*; $B_{f}$ is the corresponding bias, *s* is the stride of convolution operation, and *p* represents padding. In the Conv2D layer, this operation is applied independently for each filter and each time step. This approach ensures that spatial features are captured effectively at each time step, laying the foundation for the subsequent ConvLSTM2D layer to process these features along with their temporal dynamics.

**Figure 1:**
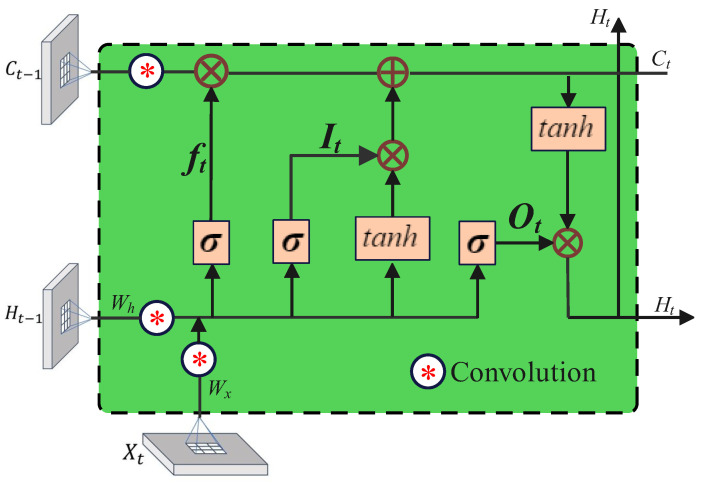
Structure of the ConvLSTM2D layer.

The ConvLSTM2D layer is a type of recurrent layer that combines convolutional and LSTM capabilities. It is applied to capture spatiotemporal features, thereby improving the model’s forecasting accuracy and computational efficiency. The architecture of the ConvLSTM2D layer is shown in Figure 1. This unique combination of layers in our model enhances its ability to effectively handle sequential data that demonstrates spatial dependency, especially images presented in a temporal sequence. The activation functions tanh and hard-sigmoid are utilized to regulate non-linearity and the flow of information within a neural network. The utilization of dropout and recurrent dropout techniques serves to alleviate the issue of overfitting, whereas the implementation of batch normalization supports the stability of activations. The mathematical formulation for the ConvLSTM2D layer is represented in the following manner:

(8) $$\begin{array}{cc} i_{t} & =\sigma (X_{t}^{\prime }*W_{xi}+H_{t-1}*W_{hi}+b_{i}), \end{array}$$

(9) $$\begin{array}{cc} f_{t} & =\sigma (X_{t}^{\prime }*W_{xf}+H_{t-1}*W_{hf}+b_{f}), \end{array}$$

(10) $$\begin{array}{cc} g_{t} & =\tanh(X_{t}^{\prime }*W_{xg}+H_{t-1}*W_{hg}+b_{g}), \end{array}$$

(11) $$\begin{array}{cc} o_{t} & =\sigma (X_{t}^{\prime }*W_{xo}+H_{t-1}*W_{ho}+b_{o}), \end{array}$$

(12) $$\begin{array}{cc} C_{t} & =f_{t}⊙C_{t-1}+i_{t}⊙g_{t}, \end{array}$$

(13) $$\begin{array}{cc} H_{t} & =o_{t}⊙\tanh\left(C_{t}\right). \end{array}$$

The ConvLSTM2D model utilizes various variables and operations. Specifically, $X_{t}^{\prime }$ denotes the input at time *t*, $H_{t}$ represents the hidden state, $C_{t}$ indicates the cell state, *W* is weight matrices, *b* is the bias vector, ∗ denotes the convolution operation, ⊙ represents element-wise multiplication, and σ denotes the sigmoid function. Equations (8) and (9) calculate the activation of the input gate $i_{t}$ and forget gate $f_{t}$, respectively. The calculation of $i_{t}$ and $f_{t}$ involves the evaluation of $X_{t}^{\prime }$, the previous hidden state $H_{t-1}$, and respective bias terms $b_{i}$ and $b_{f}$ using the convolution operation. $i_{t}$ determines the specific information that needs to be updated within the cell state. The extent to which the previous cell state $C_{t-1}$ should be disregarded is determined by $f_{t}$. The candidate values $g_{t}$ in Equation (10) are obtained by combining $X_{t}^{\prime }$, $H_{t}-1$, and a bias term $b_{g}$ with the hyperbolic tangent function tanh. The resultant values have been restricted to stay within the range of −1 and 1, thanks to the hyperbolic tangent function. LSTM can selectively store and use the necessary data for future predictions in a sequence by using the calculation to determine the new information that might be added to the cell state. In Equation (11), the activation of $o_{t}$ is responsible for the amount of data from the current cell state that should be output. The ratio of information to be passed to the output is calculated using the sigmoid function sigma, which reduces the values between 0 and 1. Equation (12) calculates the updated $H_{t}$ by performing an element-wise multiplication between $o_{t}$ and the hyperbolic tangent of the modified $C_{t}$. The concealed state is subsequently utilized for making predictions in the future or transmitted to the subsequent time step in a sequence. Finally, by applying the output gate output $o_{t}$ element-wise multiplied by the hyperbolic tangent of the updated $C_{t}$, Equation (13) estimates the new hidden state $H_{t}$. The following predictions are made using this hidden state, or it is transferred to the following time step in a sequence. The integration of convolutional and LSTM-like operations within the ConvLSTM2D layer facilitates the model’s ability to analyze intricate spatiotemporal patterns, thereby enhancing the accuracy of weather predictions. Further details about layers of the proposed methodology are given in Table 1.

**Figure 2:**
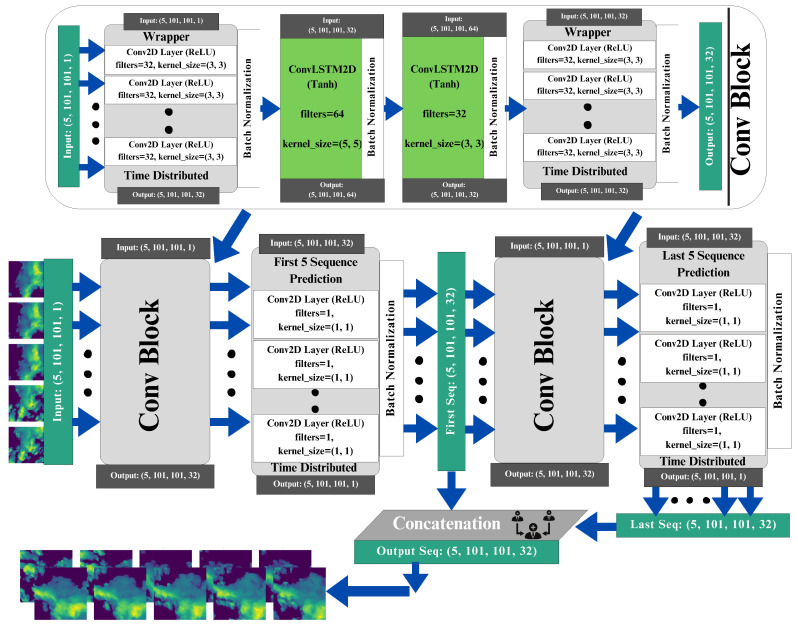
The overall architecture of the ConvLSTM2D diagram.

A graphical representation of the proposed methodology can be seen in Figure 2. The ConvLayers function is designed to handle input sequences by passing them through a series of layers, which utilize Conv2D and ConvLSTM2D operations to extract spatial and temporal features. The initial ConvLayers operation employs 32 filters with a convolutional kernel size of 3×3, batch normalization is used to normalize activations, and the second layer incorporates 64 filters, a convolutional kernel of size 5×5, and padding. The third layer uses 32 filters and different dropout values, and batch normalization is applied again. The final layer comprises a time-distributed Conv2D operation, similar to the initial layer but containing 64 filters. The input layer is characterized by the input sequence, with dimensions of batch_size (5, 101, 101, 1). The initial layer uses the Convblock function to handle the input sequence, effectively capturing spatial and temporal characteristics. The next layer predicts the middle of the sequence using a time-distributed Conv2D operation, followed by batch normalization. The features obtained from the middle prediction are further refined by subjecting its output to the ConvLayers method. The output sequence is predicted by the final layer using a time-distributed Conv2D operation employing a 1×1 convolutional kernel and sigmoid activation. The concatenation of the predictions for the middle and output sequences results in a final output sequence that includes information from both predictions. The model effectively utilizes a combination of kernel sizes, including (3, 3), (5, 5) and (1, 1), allowing it to capture both local and global features in spatiotemporal data. This architecture offers a comprehensive and resilient approach to precipitation nowcasting through the efficient utilization of Conv2D and ConvLSTM2D operations with a wide range of kernel sizes. Further comprehension of the details can be found in Algorithm 1.
**Algorithm 1** Pseudocode for the ConvLSTM2D model for sequence data processing.  1:**function** ConvLayers(input_seq, pattern = “1”)  2:     *x* ← Conv2D(input_seq, 32, (3, 3), ReLU, padding = ‘same’)  3:     *x* ← BatchNorm(*x*)  4:     *x* ← ConvLSTM2D(*x*, 64, (5, 5), tanh, hard_sigmoid, glorot_uniform)  5:     *x* ← BatchNorm(*x*)  6:     *x* ← ConvLSTM2D(*x*, 32, (3, 3), tanh, hard_sigmoid, glorot_uniform)  7:     *x* ← BatchNorm(*x*)  8:     *x* ← Conv2D(x, 32, (3, 3), ReLU, padding = ‘same’)  9:     *x* ← BatchNorm(*x*)10:     **return** *x*11:**end function**

Compared with existing methods of precipitation nowcasting, the ConvLSTM2D model has a number of clear advantages. It excels at spatial-temporal integration, fusing together spatial data from radar imagery with temporal dependencies in weather data to give a thorough understanding of weather patterns. Additionally, by combining ConvLSTM2D in an original way, the model is able to capture complex dependencies and relationships, which leads to an improvement in prediction accuracy when compared with conventional methods. It is noteworthy that the model’s architecture is highly adaptable and can be tailored to suit particular weather forecasting tasks, demonstrating its adaptability. It is a practical option for real-time precipitation nowcasting because it balances accuracy and efficiency while maintaining its advanced capabilities at a reasonable computational cost.

## 5. Experimental Setup

### 5.1. Evaluation Dataset

We used the CIKM AnalytiCup 2017 Competition dataset for experiments in this study. Researchers and data scientists find this dataset difficult due to noise and sparse radar reflectivity values, especially in lower atmospheric layers and oceanic regions. Precipitation nowcasting is complicated by rainfall patterns requiring advanced modeling. The dataset also has a significant weather imbalance, with most areas having zero or very low weather. Traditional deep-learning models may struggle with heavily skewed classes, complicating modeling. Researchers and data scientists must use advanced data processing, feature extraction, and deep-learning methods to address these issues. Preprocessing noisy and sparse radar reflectivity data, engineering relevant features, and developing robust deep-learning models for imbalanced data require advanced methods.

**Figure 3:**
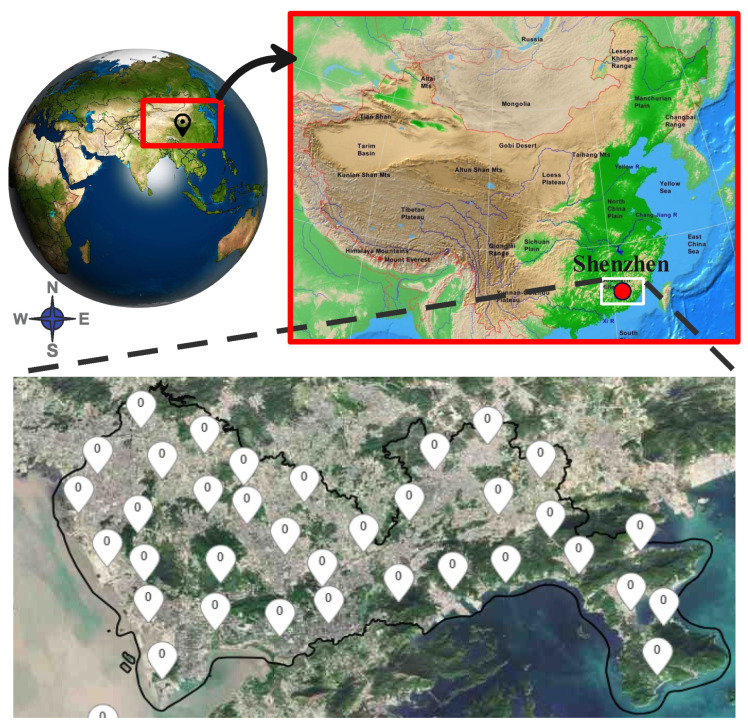
The study region: top—Shenzhen, China’s location (source: Google Maps); bottom—Weather observation sites in Shenzhen (source: Meteorological Bureau of Shenzhen Municipality’s website (http://weather.sz.gov.cn/en/en_shenzhentianqi, accessed on 1 December 2023)).

The dataset used in this study consists of authentic radar correlates and precipitation observations sourced from meteorological distribution centers. The objective is to forecast the weather at a specified location within a time frame ranging from 1 to 2 h in the future. This prediction is made by analyzing radar images of the target site and surrounding area, considering various temporal and altitudinal factors. The dataset is made up of multiple dimensions, including a central target site on each radar map, precipitation measurements for the target site, 15-time intervals with a duration of 6 min each, four height levels with a 1 km difference between them, and a grid of 101×101 structures covering an area of 101×101 square kilometers surrounding the target site as shown in Figure 3. The model uses a dataset divided into three sets for training, validation, and testing. The training set contains 8000 samples, providing diverse data for learning. The validation set has 2000 samples, serving as a benchmark for the model’s performance on unexplored data. The test set has 4000 samples, assessing the model’s generalization and predictive capabilities.

**Figure 4:**
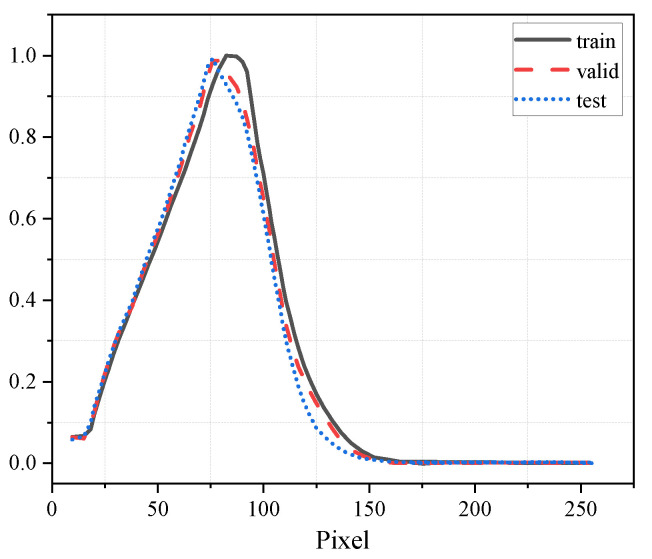
Histograms: non-zero pixel values in the dataset.

The input images employed in the model undergo a standardization procedure to guarantee consistency and suitability with the neural network structure. The images in the challenge have been adjusted to a specific size of 101 × 101 pixels, with each image containing only one channel. Each pixel in the dataset has a value between 0 and 255, corresponding to an area of 1 km × 1 km. Figure 4 illustrates the pixel distribution across the training, validation, and testing datasets. The noticeable difference in distribution between the training and testing datasets highlights the challenges inherent in the task of nowcasting.

Each test sample contains 15 radar images captured at 6 min intervals, crucial for capturing weather patterns’ dynamic evolution. The model’s design uses the initial five images as input sequences, setting the context for prediction, and the subsequent 10 images as output sequences. This segmentation aligns with the model’s objective of predicting images for 1 h based on a 30 min input.

### 5.2. Data Preprocessing

**Data Preparation:** A preprocessing step has been performed on the CIKM dataset to remove irrelevant features and sanitize the data. In the specific context of precipitation nowcasting, this process may entail the selection of key variables such as radar reflectivity, precipitation intensity, and spatiotemporal coordinates.

**Data Normalization:** Deep learning models are sensitive to data scaling. Thus, prior to training, all radar echo map data are normalized to the range [−1, 1] as per the following equation:

(14) $$x_{norm}=(b-a)\frac{x-\min(x)}{\max(x)-\min(x)},$$

where $x_{norm}$ represents the normalized data, *a* and *b* are constants used to scale the data within the desired range, and max(x) and min(x) are the maximum and minimum values in the dataset, respectively.

### 5.3. Training Setting

**Figure 5:**
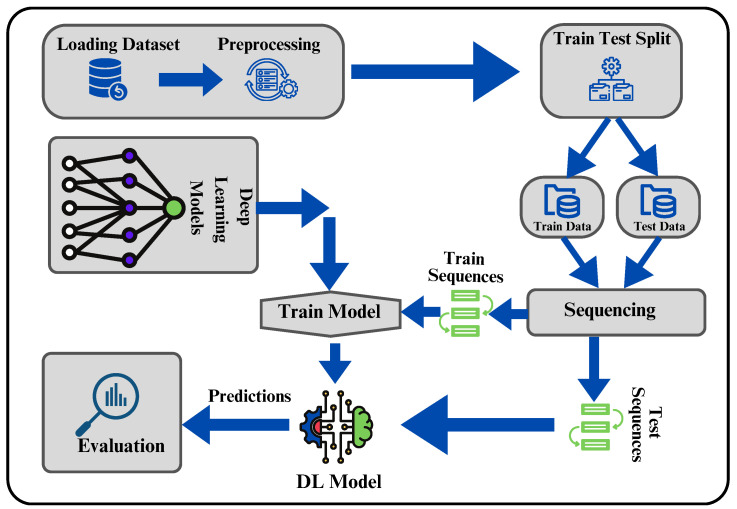
Diagram illustrating the execution of the proposed ConvLSTM2D model.

Our model undergoes fine-tuning with a composite loss function that incorporates both L1 and L2 regularization techniques. During the training phase, the model is optimized using the ADAM optimizer [34] with a learning rate of 0.001. The experimental implementation is conducted using the TensorFlow framework and executed on an NVIDIA GeForce RTX GPU. The model’s execution can be viewed in graphical form in Figure 5.

### 5.4. Evaluation Criteria

Following previous studies, a transformation is applied to every pixel value in the input data. The conversion process is executed by means of the following mathematical equation.

(15) $$\mathrm{dBZ}=\frac{\mathrm{pixel}\_\mathrm{value}\times 95}{255}-10.$$

This transformation modifies the pixel values in order to improve their appropriateness for subsequent processing. During the evaluation process, a threshold procedure is applied to both the predicted echo map and the ground truth echo map. Pixels that have values surpassing the specified threshold are assigned a value of 1, whereas pixels with values below the threshold are assigned a value of 0. The process of converting binary values allows for the evaluation of model performance using four distinct categories: true positives (TP), false positives (FP), true negatives (TN), and false negatives (FN). This evaluation process utilizes three distinct thresholds, namely 5 dBZ, 20 dBZ, and 40 dBZ. The utilization of these thresholds facilitates a comprehensive evaluation of the model’s capacity to differentiate between different levels of intensity in the echo maps. Furthermore, the Heidke skill score (HSS) and critical success index (CSI) metrics are computed to evaluate the results. The mathematical expressions for HSS and CSI are as follows:

(16) $$\mathrm{HSS}=\frac{2(\mathrm{TP}\times \mathrm{TN}-\mathrm{FN}\times \mathrm{FP})}{\left(\mathrm{TP}+\mathrm{FN})(\mathrm{FN}+\mathrm{TN})+(\mathrm{TP}+\mathrm{FP})(\mathrm{FP}+\mathrm{TN}\right)},$$

(17) $$\mathrm{CSI}=\frac{\mathrm{TP}}{\mathrm{TP}+\mathrm{FN}+\mathrm{FP}}.$$

In addition, the mean absolute error (MAE) and structural similarity index (SSIM) metrics are employed to assess the proposed methodology and alternative approaches. MAE represents the arithmetic mean of the absolute differences between the actual and predicted values. The mathematical expression used to compute MAE is as follows:

(18) $$\mathrm{MAE}=\frac{1}{n}\sum_{i=1}^{n}\left|y_{i}-{\hat{y}}_{i}\right|$$

where *n* represents the total number of observations, $y_{i}$ denotes the actual value of a specific observation, and ${\hat{y}}_{i}$ represents the predicted value of the identical observation. SSIM is a numerical metric used for measuring the degree of structural similarity between an image under evaluation and a reference image. The analysis has been built upon the three fundamental elements encompassing intensity, contrast, and structure within an image. The mathematical expression used to compute SSIM is as follows:

(19) $$\mathrm{SSIM}\left(x,y\right)=\frac{\left(2\mu_{x}\mu_{y}+c_{1}\right)\left(2\sigma_{xy}+c_{2}\right)}{\left(\mu_{x}^{2}+\mu_{y}^{2}+c_{1}\right)\left(\sigma_{x}^{2}+\sigma_{y}^{2}+c_{2}\right)}.$$

In the given equation above, $\mu_{x}$ and $\mu_{y}$ represent the average intensities of images *x* and *y*, respectively. In a comparable manner, $\sigma_{x}^{2}$ and $\sigma_{y}^{2}$ represent the variances of images *x* and *y*, while $\sigma_{xy}$ indicates the covariance between images *x* and *y*. Additionally, the constants $c_{1}$ and $c_{2}$ are added to prevent division by zero.

## 6. Results and Discussions

### 6.1. Comparison Studies

**Figure 6:**
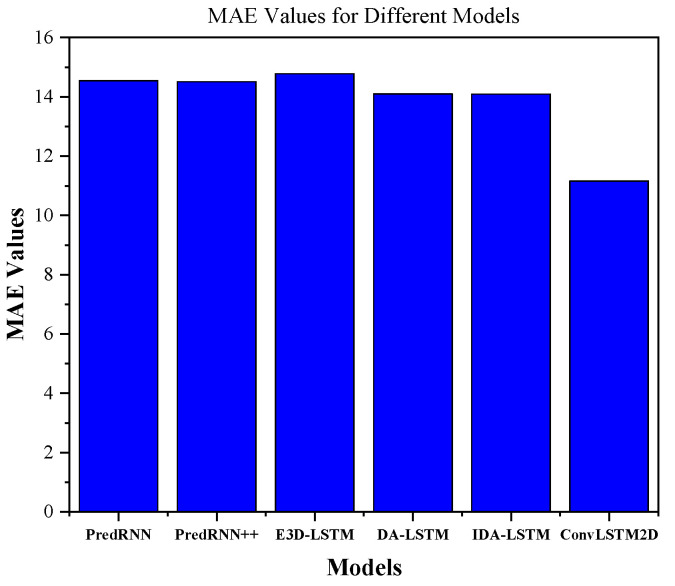
MAE comparison results.

**Figure 7:**
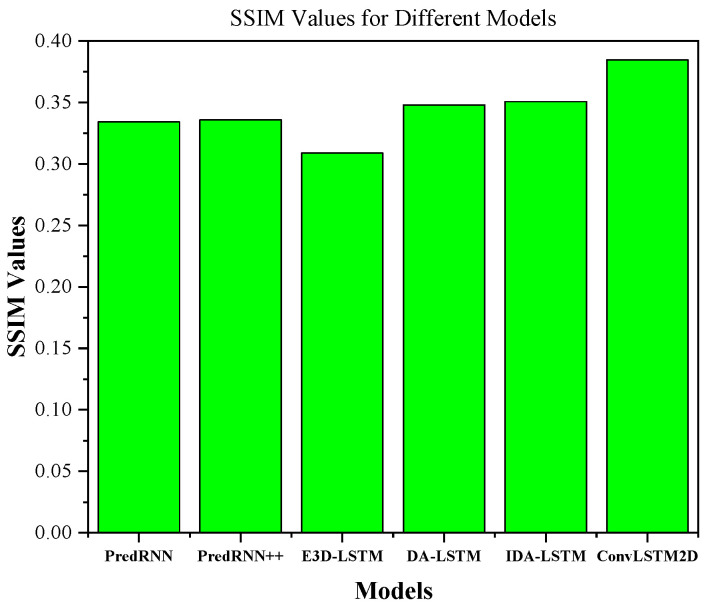
SSIM comparison results.

**Figure 8:**
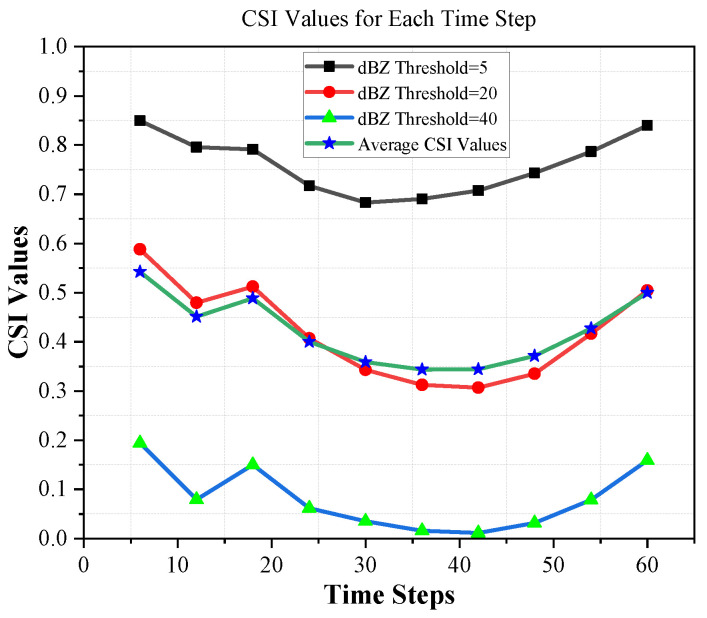
CSI results of ConvLSTM2D for thresholds 5, 20, 40 and average per time step.

**Figure 9:**
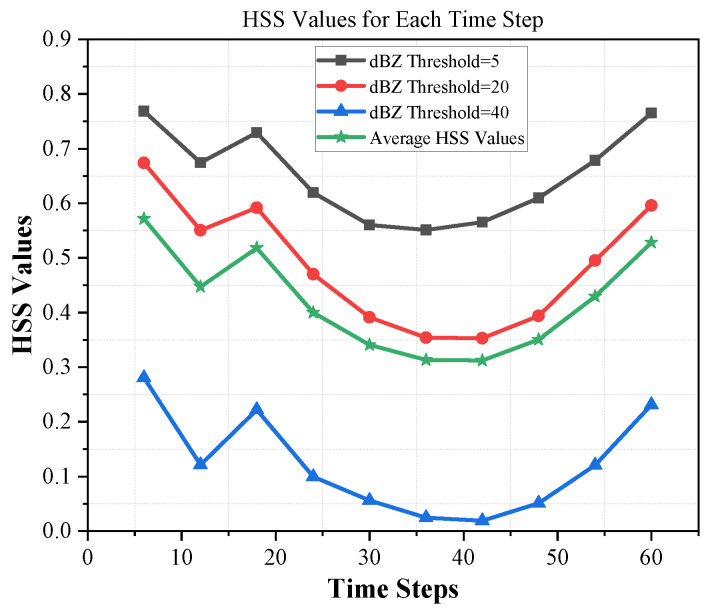
HSS results of ConvLSTM2D for thresholds 5, 20, 40 and average per time step.

Table 2 shows the performance comparison of ConvLSTM2D with different dBZ threshold parameter settings (5, 20, and 40). The “avg” column shows the average performance. The evaluation of the methods is conducted based on four metrics, namely HSS, CSI, MAE, and SSIM. According to Table 2, it can be observed that the proposed ConvLSTM2D demonstrates superior performance compared with all other methods in terms of HSS, CSI, and SSIM. It also implies that ConvLSTM2D has higher accuracy, greater success, and more comparable characteristics to the observed weather than the other methods. Furthermore, the ConvLSTM2D model outperforms the other methods significantly in terms of MAE, indicating its exceptional accuracy in predicting weather intensity when compared with the alternative approaches. This observation underscores ConvLSTM2D’s superior capability in capturing both minimal patterns and low-level intricacies of weather prediction compared to alternative methodologies. For further clarification, the results of MAE and SSIM are also graphically shown in Figure 6 and Figure 7. ConvLSTM2D’s performance in terms of CSI and HSS is depicted in Figure 8 and Figure 9 across various time steps. These plots are specifically provided for different dBZ threshold levels, including 5, 20 and 40, offering a thorough understanding of the model’s effectiveness in these distinct intervals.

The possible reason for the notable effectiveness of ConvLSTM2D lies in its utilization of a convolutional LSTM layer to represent the temporal dynamics associated with radar echo images. This approach allows for the capture of detailed and non-linear precipitation patterns. Unlike ConvLSTM2D, alternative approaches such as PredRNN [16] and E3D-LSTM [15] employ separate architectures, which might exhibit limitations in effectively capturing the temporal dependencies and variations inherent in radar echo images. Another potential explanation is that ConvLSTM2D employs multiple layers of convolutional LSTM, enabling the model to acquire high-level characteristics of the radar echo images at various levels of abstraction. The alternative approaches employ a limited number of convolutional LSTM layers and potentially lack the capacity to effectively capture specific characteristics within these images. Hence, drawing from the findings presented in Table 2, it can be inferred that ConvLSTM2D shows competitiveness and effectiveness as a technique for precipitation nowcasting compared with the state-of-the-art. Our model demonstrates an impressive ability to accurately predict weather in the immediate future, achieving a high level of success and similarity. These findings emphasize the potential of ConvLSTM2D as a promising and dependable method for weather prediction, thereby highlighting its capacity to offer precise and timely predictions for current weather terms.

### 6.2. Visualization of Prediction Results

**Figure 10:**
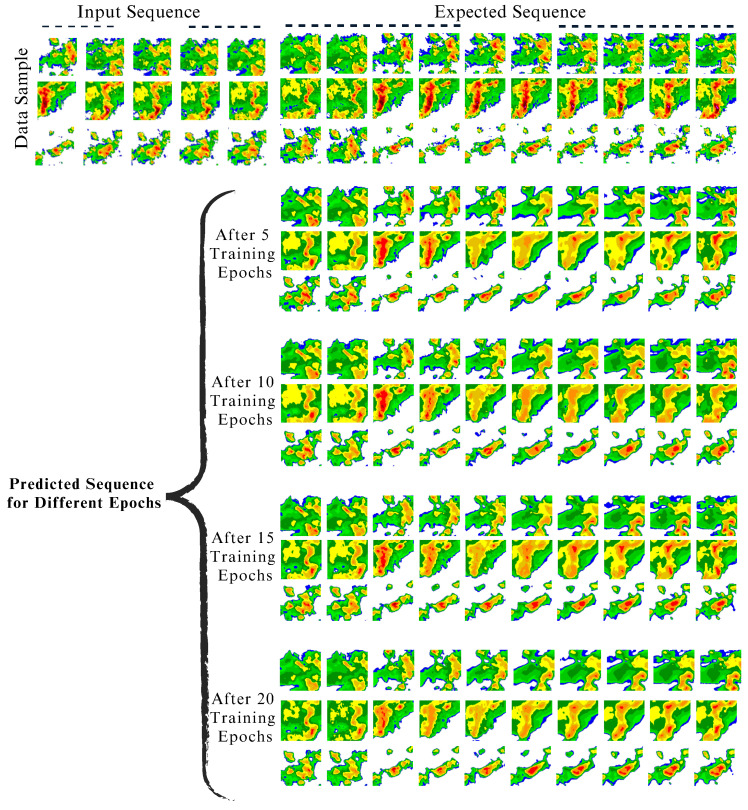
ConvLSTM2D results on the CIKM dataset: top—15 sequence data samples (5 input sequences, 10 expected output sequences); bottom—predicted output sequences across epochs.

**Figure 11:**
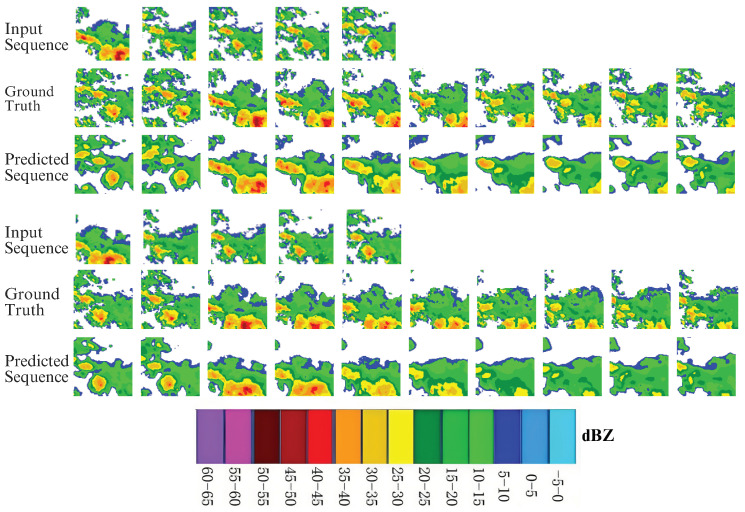
Visualization of the predicted radar echo maps using ConvLSTM2D.

Further, the ConvLSTM2D model uses a temporal sequence of images that are separated from one another by 6 min. The model’s input involves a sequential arrangement of five images, influencing a predictive time frame of 30 min. Following this, the model produces predictions for a set of 10 images, which expands the projected sequence to cover a time frame of up to 60 min. Figure 10 presents the visual results of using the ConvLSTM2D model on the CIKM dataset. The upper portion of this figure displays three rows of data samples, each consisting of 15 sequences. These include five input sequences and ten corresponding expected output sequences. This visualization shows how the model processes and responds to the input images. The bottom portion of this figure displays the model’s predicted output sequences at various epochs. The post-training performance of ConvLSTM2D with optimally selected parameters is shown in Figure 11. The ability of the model to preserve areas with high echo values can be observed from the figure. Furthermore, it is observable from the ground truth sequence that regions of high echo value decrease over time, and the proposed model nicely predicts this decreasing trend.

### 6.3. Ablation Studies

The initial ConvLSTM2D architecture failed to generate favorable results, producing uniformly black predictions and struggling to learn meaningful spatial-temporal patterns. Recognizing these limitations, time-distributed Conv2D layers were introduced but this failed to bring significant improvements. Another iteration of the architecture attempted to address these limitations by introducing additional ConvLSTM2D layers and incorporating regularization techniques. However, despite the increased complexity, the results remained unsatisfactory, underscoring the challenge of balancing model complexity with the ability to generalize effectively. Then, a sequence-based design with encoder and decoder blocks fell short of expectations. The breakthrough occurred in the final version, where time-distributed Conv2D layers before ConvLSTM2D layers significantly improved results. Iterative refinement of spatial features and the inclusion of mixed kernel sizes (1,1), (3,3), and (5,5) played a crucial role in capturing fine-grained details and global patterns for accurate predictions. This strategic combination underscores the importance of adapting architecture to the unique challenges of the precipitation nowcasting task.

## 7. Conclusions

In this paper, we introduce ConvLSTM2D, a dual-component neural network architecture tailored for precipitation nowcasting. The model combines the time-distributed Conv2D layer with the recurrent ConvLSTM2D layer. The time-distributed layer executes parallel Conv2D operations on each input image, enabling effective spatial pattern analysis. The subsequent ConvLSTM2D layer combines convolutional and LSTM capabilities, dedicated to capturing spatiotemporal features. Notably, the unique combinations of these layers are used with different kernel sizes at each level to enhance the capability of the model for capturing global features in the input sequences. We validate the feasibility and correctness of the proposed ConvLSTM2D model using a real-world dataset and benchmark it against existing state-of-the-art deep learning models, consistently showcasing ConvLSTM2D’s supremacy across all evaluation metrics. Our research underscores the pivotal role of innovative model design in addressing the complexities of real-time precipitation nowcasting. ConvLSTM2D’s exceptional performance marks a significant stride toward achieving more accurate and efficient precipitation nowcasting, with vast potential applications across diverse domains.

## Figures and Tables

**Table 1:** Details of the proposed methodology: layers and parameter settings.

Layer	Kernel Size	Padding	Filters	Activation	Output Size
Time Distributed (Conv2D)	3 × 3	1 × 1	32	ReLU	5 × 101 × 101 × 32
ConvLSTM2D	5 × 5	1 × 1	64	Tanh	5 × 101 × 101 × 64
ConvLSTM2D	3 × 3	1 × 1	32	Tanh	5 × 101 × 101 × 64
Time Distributed (Conv2D)	3 × 3	1 × 1	32	ReLU	5 × 101 × 101 × 32

**Table 2:** Performance evaluation: ConvLSTM2D vs. other models.

dBZ Threshold	HSS ↑				CSI ↑				MAE ↓	SSIM ↑
	5	20	40	Avg	5	20	40	Avg		
**PredRNN [16]**	0.7080	0.4911	0.1558	0.4516	0.7691	0.4048	0.0839	0.4198	14.54	0.3341
**PredRNN++ [17]**	0.7075	0.4993	0.1574	0.4548	0.7670	0.4137	0.0862	0.4223	14.51	0.3357
**E3D-LSTM [15]**	0.7111	0.4810	0.1361	0.4427	0.7720	0.4060	0.0734	0.4171	14.78	0.3089
**DA-LSTM [14]**	0.7184	0.5251	0.2127	0.4854	0.7765	0.4376	0.1202	0.4448	14.10	0.3479
**IDA-LSTM [14]**	0.7179	0.5264	**0.2262**	0.4902	0.7752	0.4372	**0.1287**	0.4470	14.09	0.3506
**ConvLSTM2D**	**0.8063**	**0.6867**	0.1550	**0.5493**	**0.8026**	**0.6005**	0.1072	**0.5035**	**11.16**	**0.3847**

## Data Availability

Code and data to reproduce our experiments are available at https://github.com/farahnaz45/ConvLSTM2D.
